# Effects of FTY720 on brain neurogenic niches in vitro and after kainic acid-induced injury

**DOI:** 10.1186/s12974-017-0922-6

**Published:** 2017-07-24

**Authors:** Raffaela Cipriani, Juan Carlos Chara, Alfredo Rodríguez-Antigüedad, Carlos Matute

**Affiliations:** 1 Centro de Investigaciones Biomédicas en Red (CIBERNED), Achucarro Basque Center for Neuroscience and Departamento de Neurociencias, Universidad del País Vasco (UPV/EHU), E-48940 Leioa, Spain; 2 Servicio de Neurología, Hospital Universitario de Cruces, Barakaldo, Spain

**Keywords:** FTY720, Neural stem cells, Neurogenesis, Oligodendrocyte progenitor cells, Doublecortin, NG2, Kainic acid

## Abstract

**Background:**

FTY720 (fingolimod, Gilenya™) is an oral, blood-brain barrier (BBB)-passing drug approved as immunomodulatory treatment for relapsing-remitting form of the multiple sclerosis (MS). In addition, FTY720 exerts several effects in the central nervous system (CNS), ranging from neuroprotection to reduction of neuroinflammation. However, the neurogenic and oligodendrogenic potential of FTY720 has been poorly investigated. In this study, we assessed the effect of FTY720 on the production of new neurons and oligodendrocytes from neural stem/precursor cells both in vitro and in vivo.

**Methods:**

Neural stem cells (NSCs) derived from the young rat subventricular zone (SVZ) were exposed to FTY720 (10, 100 nM), and their differentiation into neurons and oligodendrocytes was measured using immunofluorescence for anti-β-III tubulin or CNPase (2′,3′-cyclic nucleotide 3′-phosphodiesterase) as markers of mature neurons or oligodendrocytes, respectively. In addition, intracerebroventricular (*icv*) administration of kainic acid (KA; 0.5 μg/2 μl) in Sprague-Dawley rats was used as an in vivo model of neuronal death and inflammation. FTY720 was applied *icv* (1 μg/2 μl), together with KA, plus intraperitoneally (*ip*; 1 mg/kg) 24 h before, and daily, until sacrifice 8 days after KA injection. To visualize cell proliferation in the hippocampus and in white matter regions, rats were administered 5-bromo-2-deoxyuridine (BrdU) 100 mg/kg, *ip* injected every 2 days. Immunohistochemical analyses were performed on rat brain slices to measure the production of new neuronal precursors (doublecortin/DCX^+^ cells) and new oligodendrocytes precursors (proteoglycan/NG2^+^ cells).

**Results:**

In this study, we observed that FTY720 increased postnatal NSCs differentiation into both neurons and oligodendrocytes in vitro. In turn, in adult animals, FTY720 enhanced the percentage of BrdU^+^ cells coexpressing DCX marker, both in basal (FTY720 alone) and in neurodegenerative (FTY720 + KA) conditions. However, FTY720 had only a partial effect on proliferation and differentiation of oligodendrocyte progenitor cell (OPC) population in vivo.

**Conclusions:**

FTY720 promotes neurogenesis and oligodendrogenesis in vitro under basal conditions. In addition, it increases the generation of neuroblasts and oligodendrocytes after excitotoxic brain injury. This suggests that FTY720 has the potential to activate the neurogenic niche and thus favour tissue repair after lesion.

**Electronic supplementary material:**

The online version of this article (doi:10.1186/s12974-017-0922-6) contains supplementary material, which is available to authorized users.

## Background

The mechanisms of adult brain repair and structural plasticity have been the subject of intensive investigation since the discovery of adult neural stem cell (NSC) niches and their ability to proliferate and differentiate into new neurons and glial cells [1, 2]. New neurons in the adult brain are produced in discrete neurogenic niches, primarily the subventricular zone (SVZ) and the subgranular zone (SGZ) in the hippocampal dentate gyrus (DG) [2, 3]. In turn, production of new oligodendrocytes in adulthood originates primarily from endogenous oligodendrocytes progenitor cells (OPCs) that are widely distributed throughout the CNS [4] and are the major source of remyelinating oligodendrocytes after demyelinating insults [5]. OPC survival/proliferation, migration to the site of injury and differentiation into mature oligodendrocytes are critical for successful remyelination and are often compromised after injury [1, 6]. Aging, as well as acute brain injury and neurodegenerative diseases, or psychiatric disorders like depression, are associated with a decline and/or dysfunction in neurogenesis [2, 7]. Thus, strategies aimed to enhance and/or redirect endogenous neurogenesis and oligodendrogenesis, e.g. through the pharmacological manipulation of the neurogenic microenvironment, represent an attractive and powerful therapeutic tool.

FTY720, a structural analogue of sphingosine, is principally known as oral drug for multiple sclerosis [8]; its bio-active form, FTY720-phosphate (FTY720-P) that mimics the structure of sphingosine-1-phosphate (S1P) and derives, in turn, from the phosphorylation by sphingosine kinase 2, is a potent S1P receptor (S1PR) modulator [9]. Interestingly, the ability of FTY720 to pass BBB and to act, following in vivo phosphorylation, through S1PRs expressed in CNS makes this drug extremely versatile, and a number of direct effects on neurons, microglia, oligodendrocytes and astrocytes have been demonstrated [10]. The regenerative capacitive of FTY720 has been observed both in vitro and in vivo. FTY720 promotes OPC proliferation and differentiation in vitro [11–13], and in vivo in experimental autoimmune encephalomyelitis (EAE) mice model [14], and increases the number of newly produced myelinating cells in a model of local demyelination induced by lysolecithin (LPC) [15] or enhances the proliferation and migration of transplanted neural progenitor cells (NPCs) in a model of viral-induced demyelination [16]. Also, a certain ability of FTY720 to induce NPC differentiation predominantly toward oligodendroglial lineage has been observed in vitro and after transplantation in mice brain [17].

FTY720 shows positive effects on survival, proliferation, migration and differentiation of NSCs in vitro [18–21]. In addition, in vivo chronic treatment with FTY720 increases proliferation, survival and formation of new neurons in healthy mouse hippocampus [19, 20], improving contextual fear memory [19] and enhanced learning and memory ability [20]. Interestingly, FTY720 treatment enhances adult neurogenesis in the hippocampal DG of mice exposed to chronic unpredictable stress [22], an experimental paradigm of depression, causing an enhanced production of BDNF and resulting in antidepressant-like effect. However, the role of FTY720 as neuro/oligogenic agent remains not fully elucidated, especially in pathological conditions. FTY720 would be good candidate to modulate the microenvironment through the production of neurothophic factors [23, 24] and to modulate the neuroinflammatory response. Moreover, S1PR signalling plays a relevant role in the regulation of neurogenesis and cellular plasticity [25, 26].

In the present study, we investigated the effects of FTY720 on the modulation of SVZ and SGZ neurogenic niches, and the production of new neurons and oligodendrocytes following injury induced by intracerebroventricular (*icv*) injection of kainic acid (KA), a glutamate receptors agonist, in rats.

## Methods

FTY720 (2-amino-2-[2-(4-octylphenyl)ethyl]propane-1,3-diol) powder was gently provided by Novartis.

### Animals

All procedures and experiments involving animals and their care were carried out according to the guidelines of the European Union Council (Directive 2010/63/EU) and Spanish regulations (Real Decreto 53/2013) on animal ethics and welfare, and under the supervision and with the approval of our internal animal ethics committee (University of the Basque Country, UPV/EHU). All possible efforts were made to minimize animal suffering and the number of animals used.

### Neurosphere cultures and differentiation assay

NSC cultures were prepared from 4 to 7-day-old Sprague-Dawley rat pups, as previously described [27, 28], with slight modifications. Briefly, the SVZ was isolated and minced with a McIlwain tissue chopper. SVZ tissue from three to four brains was digested for 7 min at 37 °C in 5 ml of trypsin/EDTA (Sigma). Digestion was stopped by adding an equal volume of trypsin inhibitor (Gibco) and 0.01% DNAse I (Sigma) for 5 min at room temperature. The cell suspension was centrifuged for 10 min at ×600*g* and the pellet mechanically dissociated 25 times in 3 ml NeuroCult^TM^ medium (STEMCELL Technologies) using a glass Pasteur pipette and 20 times using 1 ml pipette tips. The cells that remained in suspension were decanted for 20 min and the single cell suspension counted using the Neubauer method. Cells were seeded in proliferation medium [NeuroCult^TM^ medium supplemented with 10% neural stem cell factors from STEMCELL Technologies, 2 mM glutamine, penicillin/streptomycin mix, 20 ng/ml EGF/epidermal growth factor (Promega), 10 ng/ml bFGF/basic fibroblast growth factor (Promega), 10 ng/ml PEDF/pigment epithelium-derived factor (Millipore)] at a density of 10^4^cells/cm^2^ and cultivated in suspension for 7 days at 37 °C, 5% CO_2_. EGF, bFGF and PEDF were added fresh every 2–3 days. After 7 DIV (days in vitro), cells were aggregated as neurospheres. To differentiate cultures from neurons, floating neurospheres were then allowed to attach onto poly-ornithine-coated glass coverslips in 24-well plates in neuron differentiation medium (NeuroCult™ medium supplemented with differentiation factor 10× (both from STEMCELL Technologies), NGF/nerve growth factor (NGF-beta, human recombinant, #4303R-100; Biovision) and BDNF/brain-derived neurotrophic factor (BDNF, human recombinant, #4004–50; Biovision)) and differentiated for additional 7 DIV in the presence of FTY720 (reconstituted in dimethyl sulfoxide hydrochloric acid (DMSO)/50 mM HCl). Alternatively, the neurospheres were maintained for 3 days in oligodendrocyte differentiation medium, composed of DMEM supplemented with 4.5 mg/ml glucose and sodium pyruvate (Gibco), SATO (100× stock solution: 100 μg/ml BSA, 100 μg/ml transferrin, 16 μg/ml putrescine, 40 ng/ml thyroxine, 30 ng/ml tri-iodothryronine, 60 ng/ml progesterone, 40 ng/ml selenium, all of from Sigma), 6.3 mg/ml N-acetyl-cysteine (Sigma), 0.5 mg/ml insulin (Sigma), 1 μg/ml CNTF/ciliary neurotrophic factor and 10 μg/ml NT3/neurotrophin-3 (both from Peprotech). This step was considered to be the *pre-commitment* stage before oligodendrocyte differentiation. After 3 DIV, floating neurospheres were allowed to attach onto poly-ornithine-coated glass coverslips in 24-well plates in oligodendrocyte differentiation medium and differentiated for 4 DIV in the presence of FTY720.

At the end of the differentiation phase, on DIV 14, cells were fixed and immunostained for cell-specific markers of mature neurons or oligodendrocytes. The extent of differentiation both to neurons and to oligodendrocytes was evaluated in treatment conditions and control conditions as described below.

### Immunocytochemistry and evaluation of neurospheres differentiation

Following differentiation, cell cultures were fixed in 4% paraformaldehyde and permeabilized with 0.05% Triton and 5% normal goat serum in phosphate-buffered saline (PBS). Primary antibody rabbit, anti-β-III tubulin (1:300; Abcam, #Ab18207) and mouse anti-CNPase (1:500; Sigma, #C5922) were incubated overnight at 4 °C and then washed three times with 0.05% Triton in PBS. Alexa Fluor 488-conjugate secondary antibodies were incubated for 1 h in the dark at room temperature (1:500). After three washes with 0.05% Triton in PBS, cells were stained for 10 min at room temperature with propidium iodide (PI) to stain total nuclei, and further washed with PBS. Fluorescence intensity was measured using a fluorescence microplate reader equipped with appropriate excitation and emission filters to detect the fluorescent signal from Alexa 488 and PI. Differentiation was evaluated as a ratio of fluorescence intensity from β-III tubulin or CNPase-positive cells over total nuclei.

### Intracerebroventricular injection of KA, FTY720 treatment and BrdU labelling in adult rats

We used a total of 24 adult male Sprague-Dawley rats (200–250 g). Rats were kept on a 12/12 h light/dark cycle with constant ambient temperature and humidity. Food and water were available ad libitum. We used unilateral *icv* injection of KA as model of induced-seizure and neurodegeneration. Rats (*n* = 6 per experimental group) were anesthetized by *ip* of ketamine 80 mg/kg (Imalgene®, Merial Laboratorios SA) and xylazine 10 mg/kg (Rompun®, Bayer) and placed into a stereotaxic apparatus (David Kopf Instruments). KA (0.5 μg in 2 μl in saline; Abcam), alone or in combination with FTY720 (1 μg in 2 μl in saline), was injected into the right lateral ventricle (right side referred thereafter as ipsilateral), at the following coordinates from bregma: −1 mm anterioposterior, 2 mm mediolateral and 4 mm dorsoventral [29]. Injections were carried out over 5-min period using an infusion pump (KD Scientific), with a constant infusion rate of 0.4 μl/min. Animals were injected *ip* with vehicle (saline solution) or FTY720 (1 mg/kg) 24 h before *icv* injection and subsequent daily until sacrifice 8 days after KA application (see Fig. 2a for experimental design). For the *ip* injection, FTY720 was freshly prepared every day. Control animals received vehicle only. All animals received the thymidine analogue 5-bromo-2′-deoxyuridine (BrdU, Sigma, #B5002) as *ip* injection (100 mg/kg, diluted in sterile saline) every 2 days starting the day after *icv* injection, as previously used [30]. Rats were sacrificed 24 h after the last BrdU injection.

### Histology

Eight days after *icv* injection, the animals were deeply anesthetized with chloral hydrate 400 mg/kg (Panreac Quimica) and transcardially perfused with 4% paraformaldehyde in 0.1M PBS (pH 7.4). Brains were removed and immediately post-fixed in the same solution for 3 h. Then, they were washed and stored at +4 °C in PBS/Azide (0.02%) until sectioning. For all brains, series of 40-μm-thick coronal sections at the level of lateral ventricles and dorsal hippocampus were cut on a vibratome (HM 650V, Microm International) and used for immunohistology on free-floating sections, as hereinafter described. HCl antigen retrieval was used for BrdU immunostaining: before blocking step, sections were incubated in 2N HCl at 37 °C for 30 min to denature DNA, followed by two 10-min rinses in 0.1M sodium tetraborate pH 8.5 at room temperature to neutralize HCl and then rinsed twice with PBS. Immunoperoxidase staining was used for identification of proliferating, BrdU-positive nuclei. Briefly, after quenching of endogenous peroxidase (H_2_O_2_ 0.3%) and blocking/permeabilization (4% normal goat serum and 0.1% Triton X-100 in PBS), we incubated the sections overnight at 4 °C with a rat anti-BrdU antibody (AbD Serotec, #MCA2060) diluted 1:400 in blocking buffer. Subsequently, the primary antibody was detected using biotinylated goat anti-rat secondary antibodies (1:200), followed by incubation with avidin-biotin-peroxidase complex (both from Vector Laboratories). Peroxidase activity was visualized by incubation in 3,3′-diaminobenzidine (DAB) substrate (Roche). Finally, the sections were mounted in gelatin-coated slides, dehydrated through graded alcohols, cleared with xylene and coverslipped with DPX.

To analyze the cell fate of BrdU-positive cells, we performed double immunofluorescence colocalization studies with cell-specific markers. The sections were incubated with blocking and permeabilization solution (4% normal goat serum, 0.1% Triton X-100 in PBS) for 1 h at room temperature and then incubated overnight with the primary antibodies (diluted in the same solution) at 4 °C. After washing with PBS, the sections were incubated with Alexa Fluor 488- or 594-conjugate secondary antibodies (molecular probes) diluted 1:400 in the blocking solution for 1 h at room temperature. After washing with PBS, the sections were mounted on gelatine-coated slides with ProLong® Antifade Mountant (ThermoFisher Scientific). In addition to anti-BrdU antibody, we used rabbit anti-DCX (1:1000; Abcam, #Ab18723) as a marker of new neurons and mouse anti-NG2 (1:500; Chemicon, #MAb5384) as a marker of OPCs.

Negative controls in all experiments included the omission of the primary antibodies and provided no labelling, indicating the reliability and specificity of the immunostaining.

### Image capture and cell quantification

#### Immunoperoxidase staining

Two slices per animal were analyzed representing two different levels of the dorsal hippocampus. The sections were visualized using Zeiss Axioplan 2 bright field microscope coupled to an Axiocam MRc5 digital camera (Zeiss), and representative photomicrographs of ipsilateral and contralateral DG region of the hippocampus were taken under a ×10 magnification objective. Quantitative analysis of BrdU-positive cells was performed counting the immunoreactive cells along the whole SGZ of DG in each slice under ×40 magnification objective. BrdU-positive cells were considered to be within the SGZ if they were within two cell body diameters on the border between the granular cell layer (GCL) and the hilus (see Fig. 2b) [31]. Immunoreactive cells were counted in two slices per animals, and data were plotted as the mean of BrdU-positive cells per section ± SEM.

### Colocalization analysis

To analyze cell fate of newly, BrdU^+^ cells, double-immunolabelled sections were imaged with a Fluoview FV500 confocal laser scanning microscope (Olympus) equipped with Fluoviewer software (Olympus). Positive cells were counted using confocal acquired images, and the percentage of BrdU^+^ cells colabelling with cell fate markers was determined using Z-stack maximal projection of 5–10 confocal layers spaced 2.5 μm in coordination with three-dimensional orthogonal reconstruction of confocal layers (ImageJ software, http://rsbweb.nih.gov/ij/download.html). DCX and BrdU colocalization was determined over the total ipsilateral SGZ of DG in two slices per animal corresponding to two different levels of the dorsal hippocampus, and data is presented as mean positive cells per section in the case of total BrdU^+^ cells and as percentage of DCX/BrdU double-positive cells on total BrdU^+^ cells per section. NG2 and BrdU colocalization was determined at the level of lateral ventricle and at the level of the dorsal hippocampus: the measured areas selected five fields of the corpus callosum (both at ventricle and hippocampal levels), three fields of hilus and two fields of fimbria of the dorsal hippocampus (see Figs. 4a and 5d). Data are illustrated as the average of positive cells per square millimeter (mm^2^) in the case of total BrdU^+^ cells and percentage of NG2/BrdU double-positive cells on total BrdU^+^ cells per mm^2^.

### Statistical analysis

Data are expressed as mean ± SEM. Statistical analyses were performed using Prism version 5.0 (GraphPad Software, USA). Comparisons between the two groups were analyzed using two-tailed Student’s *t* test. Comparisons among multiple groups were analyzed by one-way or two-way analysis of variance (ANOVA), as appropriate, followed by Bonferroni post hoc test. *p* ≤ 0.05 or *p* ≤ 0.01 was defined as significant or highly significant, respectively.

## Results

### FTY720 promotes differentiation of NSC cultures

**Fig. 1:**
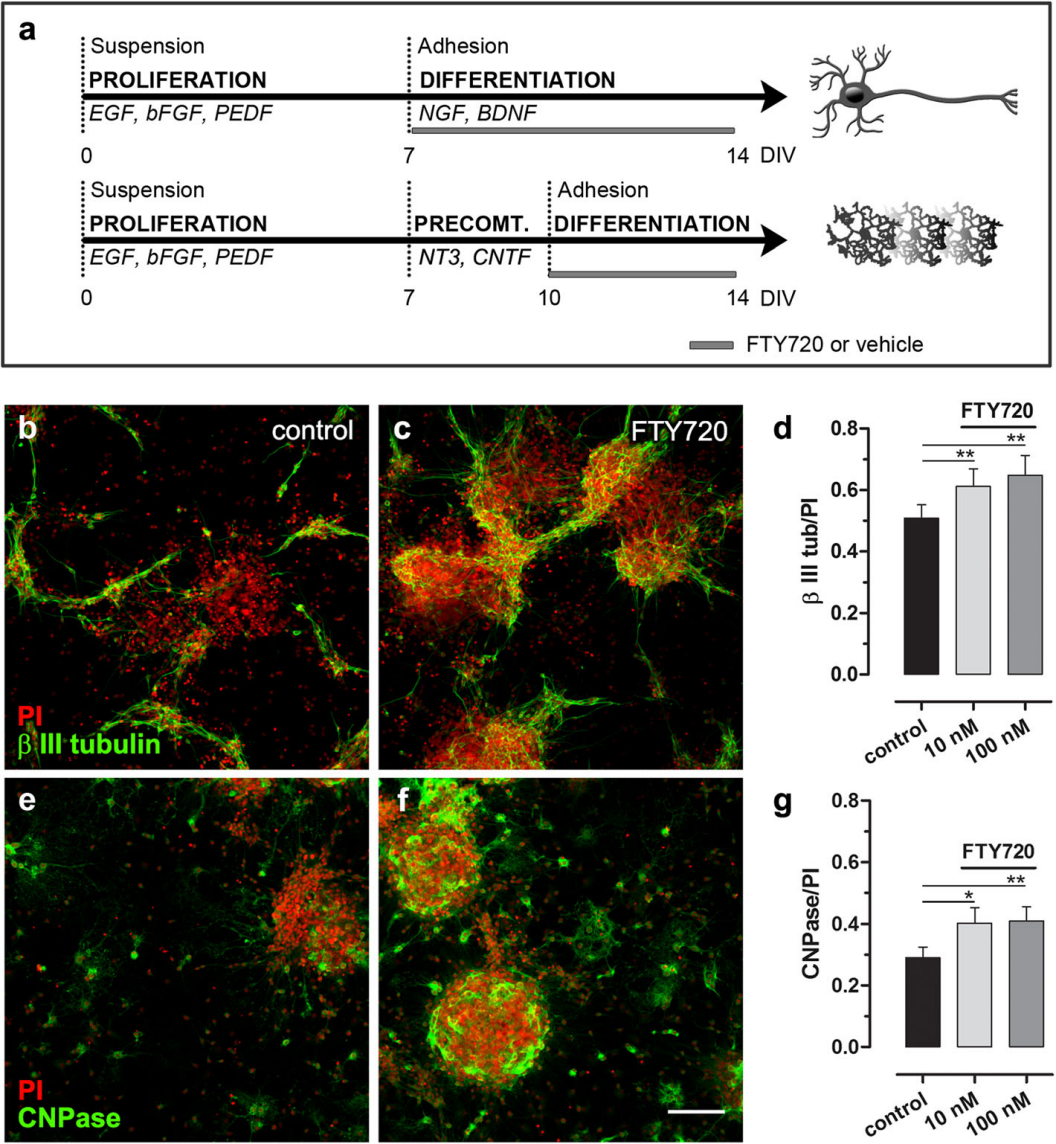
FTY720 induces differentiation of neurospheres both into neurons and oligodendrocytes*.* **a** Schematic view of the protocol used for differentiation of SVZ-derived neurospheres into neurons or oligodendrocytes. SVZ from young rats (P2–P4) was dissociated to obtain proliferating NSCs. Cells were maintained for 7 days in vitro (DIV) in the presence of the mitogenic factors EGF, bFGF and PEDF (proliferation phase). After 7 DIV, neurospheres were differentiated into neurons up to 14 days, in the presence of NGF and BDNF or pre-committed to an oligodendrocytic phenotype for 3 days and differentiated for up to 4 days in presence of NT3 and CNTF (differentiation phase). FTY720 was added to the cultures during the differentiation phase at different concentrations (10 and 100 nM). At DIV 14, cells were fixed and immunofluorescence was performed using anti-β-III tubulin (**b**, **c**) or CNPase (**e**, **f**) as markers of mature neurons or oligodendrocytes, respectively (**b**, **c** and **e**, **f**: representative photomicrographs of the staining). Total nuclei were stained with propidium iodide (PI). Fluorescence intensity both from neurons (β-III tubulin) or oligodendrocytes (CNPase) and total nuclei (PI) was measured using a fluorescence plate reader with appropriate excitation and emission filters. Differentiation was evaluated as a ratio of β-III tubulin or CNPase fluorescence intensity over total nuclei fluorescence intensity (**d**, **g**). Data are expressed as mean ± SEM of 6–7 independent experiments. Statistical analysis: paired, two-tailed Student *t* test; *p < 0.05 and **p < 0.01 vs control

We examined the neurogenic potential of FTY720 in vitro, on differentiation of NSC derived from young rat SVZ [27]. NSCs in culture proliferate and aggregate in neurospheres with multipotent stem cells properties which depending on local signals can differentiate into neurons or glial cells. In Fig. 1a, a schematic diagram of the experimental conditions is reported (see ‘Methods’ for further details). After a common phase where neurospheres proliferate as suspension cultures, they were split into two groups: neuron-oriented and oligodendrocyte-oriented differentiation cultures that were exposed to specific mitogen growth factors. At that stage, FTY720 was added in the medium at different concentrations (10 and 100 nM) to verify the ability of the drug, as compared to control conditions, to promote differentiation toward a specific cellular type. We measured the differentiation to neurons and to oligodendrocytes using immunofluorescence for β-III tubulin or CNPase, as markers of neurons or oligodendrocytes, respectively. FTY720 at either tested concentrations significantly increased NSC differentiation both into neurons (Fig. 1b–d) and oligodendrocytes (Fig. 1e–g), as compared to control conditions.

Previous studies demonstrated that neurospheres in culture express all five subtypes of S1P receptors [16], and the treatment with FTY720 was able to activate intracellular signalling cascades, indicating receptor binding and activation [16, 19, 20]. FTY720 enhanced survival, proliferation and migration of NSCs in vitro [18–21]; however, effect on differentiation remains controversial. Our results, demonstrating a positive effect of FTY720 on differentiation of postnatal NSCs, both to neurons and oligodendrocytes, indicate that FTY720 is able to accelerate neurogenesis and oligodendrogenesis from neural precursors and suggest a potential neurogenic role also in vivo. To verify that the observed effect is due to involvement of S1PRs pathways, we performed the same differentiation experiment using FTY720-P instead of FTY720, obtaining similar results (see Additional file 1: Figure S1).

### FTY720 does not alter the number of proliferating cells in SGZ after KA-induced injury in vivo

**Fig. 2:**
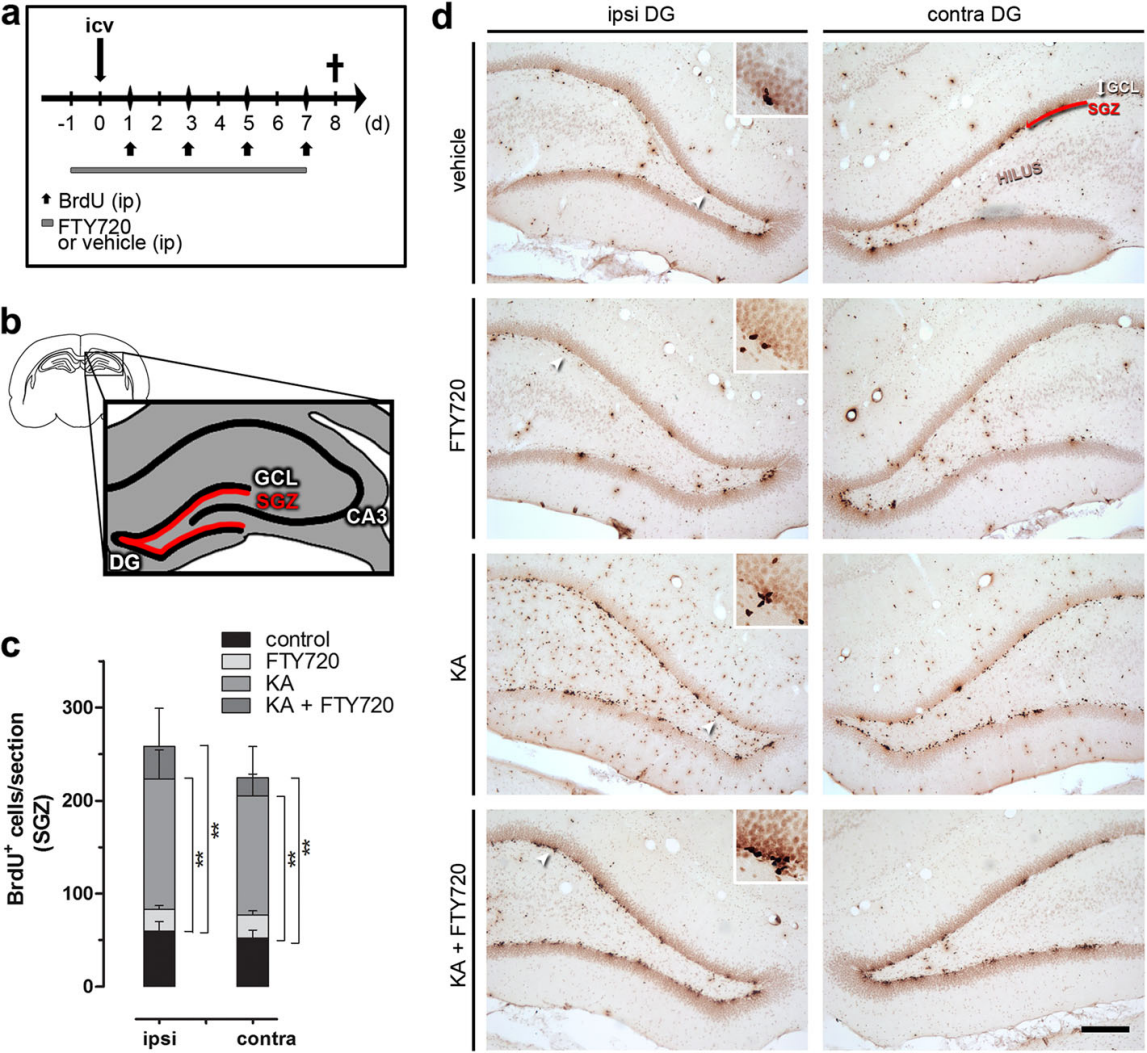
KA icv injection increases cell proliferation in dentate gyrus (DG). **a** Scheme of in vivo experimental protocol and **b** schematic diagram of the DG. Positive cells were considered to be within the SGZ if they were within two cell body diameters of the border between the GCL and the hilus. **c** Quantitative analysis of the immunoperoxidase staining for BrdU in ipsilateral (ipsi) and contralateral (contra) SGZ of the dorsal hippocampus in control, FTY720, KA and KA + FTY720-treated animals. Immunoreactive cells for BrdU were counted in two sections per rat using a bright field microscopy and a 40× objective. Data were plotted as the mean of BrdU^+^ cells per slice ± SEM. Statistical analysis: one-way ANOVA followed by Bonferroni post hoc test; **p < 0.01 vs control. Two-way ANOVA showed no significant difference between ipsilateral and contralateral BrdU^+^ cells in SGZ. *N* = 6 for each experimental group. **d** Representative images of immunoperoxidase staining of BrdU in ipsilateral and contralateral DG in all experimental groups. *Scale bar*: 200 μm

Confident with our results in vitro, we decided to explore the effect of FTY720 in adult neurogenesis in vivo, under neurodegenerative and neuroinflammatory conditions. For this aim, we investigated the effects of FTY720 in adult rat hippocampal neurogenesis following KA-induced acute neuronal death. All animals treated with KA experienced seizures in the first 2–3 h after *icv*, and we previously demonstrated that FTY720 reduced CA3 excitotoxic neuronal death and microgliosis associated with KA-induced status epilepticus [32]. We speculated whether modulation of adult neurogenesis by FTY720 could contribute to tissue repair in addition to the observed neuroprotection. To test that possibility, KA was injected in the right lateral ventricle (0.5 μg/2 μl), alone or in combination with FTY720 (1 μg/2 μl); in parallel, animals also received *ip* injection of FTY720 (1 mg/kg) or vehicle, from day −1 every day until 24 h before sacrifice (Fig. 2a). Animals were split into a total of four experimental groups: *control* (animals receiving vehicle *icv* and *ip*), *FTY720* (animals receiving FTY720 *icv* and *ip*), *KA* (animals receiving KA *icv* and vehicle *ip*) and *KA + FTY720* (animals receiving KA and FTY720 *icv* and FTY720 *ip*). Also, all animals were treated with BrdU (100 mg/kg, ip), starting from post-injection day 1 and every second days until 24 h before sacrifice (Fig. 2a), to label all proliferative cells at the S phase of cell cycle. Eight days after *icv* injection of KA, animals were sacrificed and immunohistochemistry for BrdU performed on 40-μm-thick brain slices at the level of dorsal hippocampus. The number of BrdU^+^ cells was counted in the SGZ of the DG, to estimate the effect of FTY720 on the proliferation of NSCs in adult rats in basal and neurodegenerative conditions. Positive cells were considered to be within SGZ if they were within two cell body diameters on the border between the GCL and the hilus (Fig. 2b). Figure 2d shows representative photomicrographs of BrdU staining in ipsilateral and contralateral DG (at dorsal hippocampus level), whereby GCL and SGZ are indicated by arrows. Quantitative analysis of the number of BrdU^+^ cells in SGZ revealed that KA induced a significantly increase in proliferation both in ipsilateral and contralateral hippocampus (Fig. 2c), as reported in previous studies [2, 31]. FTY720, alone or in combination with KA, slightly enhanced the number of positive cells, but the increase was not statistically significant (Fig. 2c). No significant differences were observed between the ipsilateral and contralateral hemispheres. Subsequent analyses were then performed only in the ipsilateral hemisphere. BrdU^+^ cells in GCL were also counted, but no significant differences were observed between groups (data not shown). Data obtained with BrdU immunolabelling were further confirmed by immunohistochemistry for the proliferation marker Ki67 (a nuclear protein expressed in active dividing cells) (Additional file 2: Figure S2).

### FTY720 increases the number of new neuroblasts in SGZ after KA-induced toxicity in vivo

**Fig. 3:**
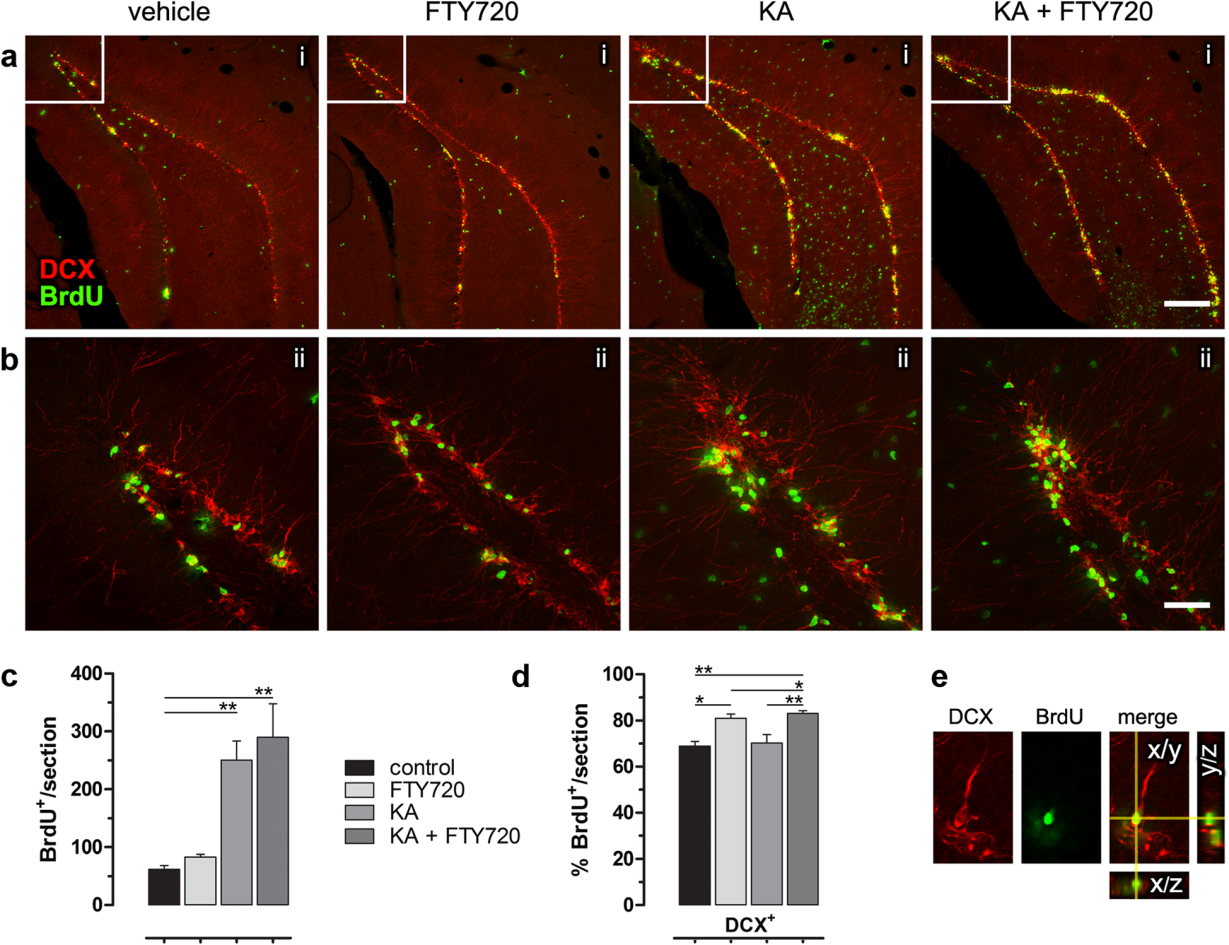
Treatment with FTY720 increases the number of DCX-positive cells in SGZ*.* **a**, **b** Representative images acquired using confocal laser scanning microscopy of double immufluorescence staining for BrdU and DCX in ipsilateral DG in saline-injected control (vehicle), FTY720, KA and KA + FTY720 injected animals, 8 days after surgery. (i) Low magnification and (ii) Z-stack maximal projection of 5–10 confocal layers spaced by 2.5 μm of the area marked with a white frame in (i). *Scale bar* (i) 200 μm and (ii) 50 μm. **c**, **d** Quantitative analysis of BrdU^+^ and DCX^+^/BrdU^+^ cells within the SGZ of the ipsilateral dorsal hippocampus. Positive cells were counted using confocal acquired images over the total SGZ of the ipsi DG in two sections per animal. DCX and BrdU colocalization was determined examining three-dimensional orthogonal reconstruction of confocal layers (ImageJ software). Data are presented as **c** mean of positive cells per slice ± SEM and **d** percentage of DCX^+^ cells over BrdU^+^ total cells. Statistical analysis: one-way ANOVA followed by Bonferroni post hoc test; *p < 0.05, **p < 0.01. *N* = 6 for each experimental group. **e** Representative confocal Z-stack images showing BrdU and DCX colocalization, as single and merged channels

To establish the nature of newborn cells in SGZ, we carried out double immunofluorescence for BrdU and DCX, a microtubule-associated protein expressed by neuronal precursor cells and immature neurons (Fig. 3a, b) and quantified BrdU and BrdU/DCX double-positive cells in the ipsilateral SGZ. Consistent with the results described above by immunohistochemistry (Fig. 2c), no difference was observed between KA and KA + FTY720-treated group (Fig. 3c) in terms of total BrdU^+^ cells, whereas FTY720 significantly increased the percentage of BrdU cells coexpressing DCX marker, both in basal (FTY720 alone) and in after injury (KA + FTY720) conditions (Fig. 3d), indicating a role for this drug in promoting new neuroblasts formation in the hippocampus. The same results were obtained when FTY720 was applied *ip* only, as reported in Additional file 3: Figure S3.

### FTY720 has only a partial effect on OPC pool in vivo after KA-induced injury

**Fig. 4:**
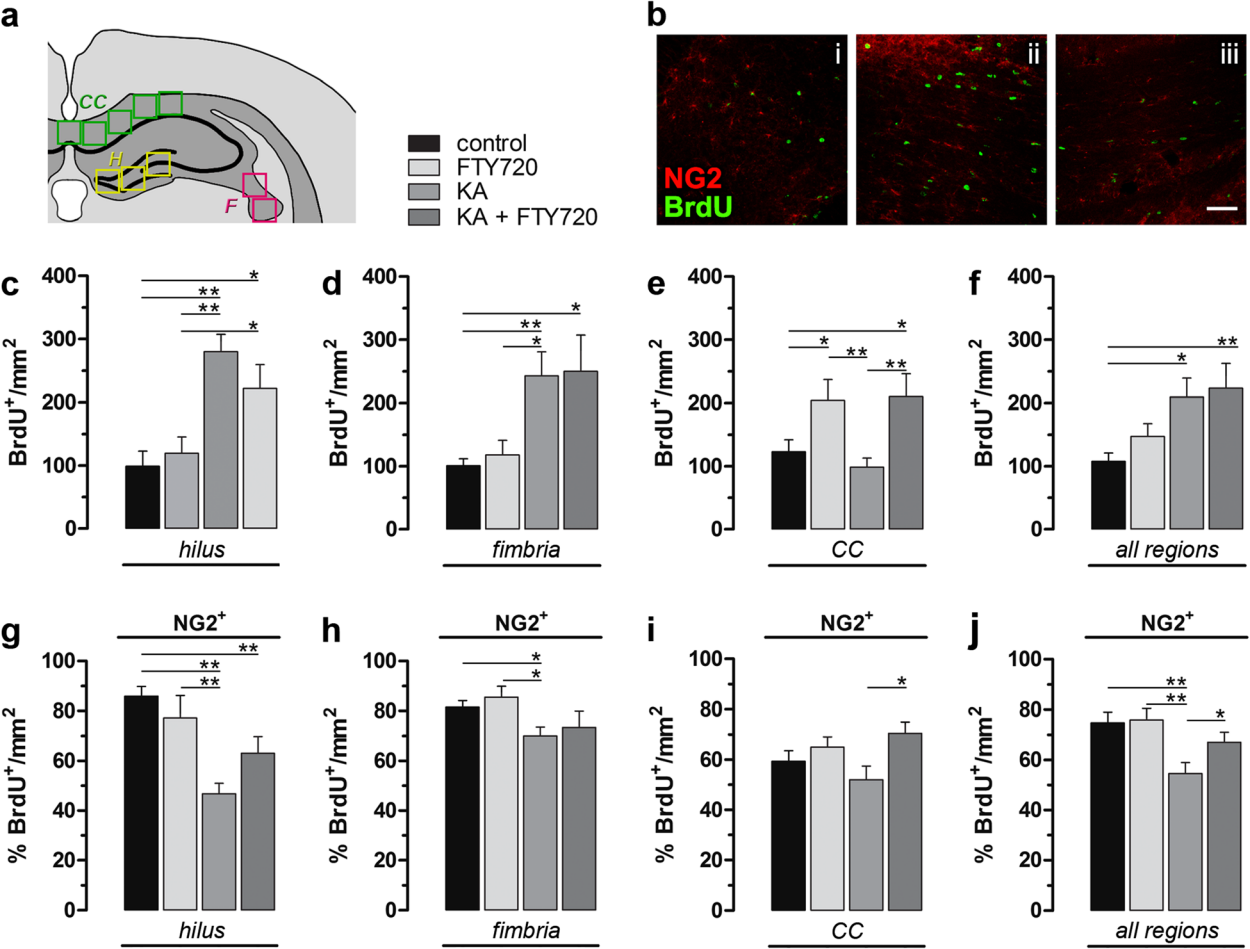
Effect of FTY720 treatment on the number of NG2^+^ cells at the level of the dorsal hippocampus*.* **a** Schematic diagram of measured areas in the ipsilateral hemisphere (dorsal hippocampus level), in hilus (H), fimbria (F) and corpus callosum (CC). **b** Representative Z-stack maximal projection of immufluorescence staining for BrdU and NG2 in the (i) hilus, (ii) fimbria and (iii) corpus callosum (KA + FTY720 group). *Scale bar*: 50 μm. **c**–**j** Quantitative analysis of BrdU^+^ and NG2^+^/BrdU^+^ cells. NG2 and BrdU colocalization was determined examining three-dimensional orthogonal reconstruction of confocal layers (ImageJ software). Data are presented as **c**–**f** mean of positive cells per mm^2^ ± SEM and **g**–**j** percentage of NG2^+^ cells over BrdU^+^ total cells per mm^2^. Statistical analysis: unpaired, two-tailed Student *t* test; *p < 0.05, **p < 0.01. *N* = 4 animals for each experimental group

**Fig. 5:**
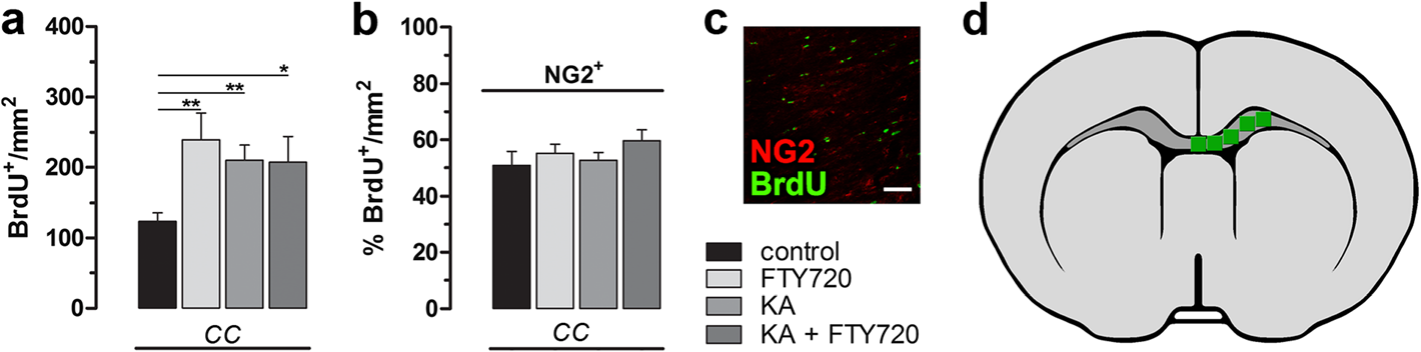
Treatment with FTY720 does not affect the number of NG2^+^ cells at the level of lateral ventricle. **a**, **b** Quantitative analysis of BrdU^+^ and NG2^+^/BrdU^+^ cells. Positive cells were counted using confocal acquired images in the corpus callosum at the level of lateral ventricle (ipsilateral hemisphere). NG2 and BrdU colocalization was determined examining three-dimensional orthogonal reconstruction of confocal layers (ImageJ software). Data are presented as **a** mean of positive cells per mm^2^ ± SEM and **b** percentage NG2^+^ cells over BrdU^+^ total cells per mm^2^. Statistical analysis: unpaired *t* test, two-tailed. *N* = 4 for each experimental group. **c** Representative double Z-stack maximal projection of immufluorescence staining for BrdU and NG2 in the corpus callosum (KA + FTY720 group). *Scale bar*: 50 μm. **d** Schematic diagram of measured areas in white matter ipsilateral hemisphere (lateral ventricle level)

FTY720 exerts direct effects on oligodendrocytic lineage via S1P receptors modulation, promoting survival, differentiation and remyelination [11, 12, 14, 33]. We tested the hypothesis that FTY720 could enhance oligodendrogenesis in vivo, promoting OPC proliferation and differentiation into mature oligodendrocytes after a brain injury caused by *icv* injection of KA, as oligodendrocytes are also vulnerable to excitotoxic insults [34]. Thus, we quantified newborn BrdU^+^ cells with an oligodendrocytic fate using the OPC marker NG2, at two different brain levels in order to cover a wider brain area: the ipsilateral corpus callosum (CC), fimbria and hilus at the level of the dorsal hippocampus and the ipsilateral CC at level of the lateral ventricles (Fig. 4a and 5d). Figure 4 summarizes the results obtained at the level of the dorsal hippocampus in term of total BrdU^+^ cells per mm^2^ (Fig. 4c–f), and percentage of NG2/BrdU double-positive cells over total BrdU^+^ cells per mm^2^ (Fig. 4g–j), in hilus, fimbria and CC, separately and all together. Percentage of newborn NG2^+^ cells has a tendency to decrease in KA-treated animals (significantly in the hilus, fimbria and in all regions pool together), compared to control, whereas significant effect between KA-treated and (KA + FTY720)-treated group was found only in the corpus callosum and in all regions plotted together. The number of analyzed cells and *P* values resulting from all statistical analyses are showed in Tables 1 and 2, respectively.

**Table 1:** Number of total analyzed cells per experimental group at the level of dorsal hippocampus in all analyzed regions (hilus, fimbria and corpus callosum). Number of animals per group is also reported

	Number of total cells analyzed per experimental group			Number of animals per group
	Hilus	Fimbria	CC	
Control	75	80	165	4
FTY720	71	95	312	4
KA	205	182	124	4
KA + FTY720	185	191	280	4

**Table 2:** Table shows *P* values resulting from all statistical analyses reported in Fig. 4c-j

	Student *t* test: *P* values							
	Hilus		Fimbria		CC		All regions	
	BrdU^+^/mm^2^	% NG2^+^BrdU^+^	BrdU^+^/mm^2^	% NG2^+^BrdU^+^	BrdU^+^/mm^2^	% NG2^+^BrdU^+^	BrdU^+^/mm^2^	% NG2^+^BrdU^+^
Control vs FTY720	0.5626	0.3813	0.5164	0.4519	0.0381	0.3482	0.1145	0.8522
Control vs KA	*P* < 0.0001	*P* < 0.0001	0.0031	0.0226	0.314	0.2924	0.0054	0.0032
Control vs KA + FTY720	0.0119	0.0071	0.0233	0.2703	0.0327	0.0788	0.0106	0.2051
FTY720 vs KA	0.0003	0.0066	0.0146	0.0164	0.0075	0.0633	0.1017	0.0028
FTY720 vs KA + FTY720	0.036	0.2137	0.0517	0.1463	0.9047	0.3714	0.0992	0.1637
KA vs KA + FTY720	0.2302	0.0554	0.9165	0.6478	0.0066	0.013	0.7783	0.0472

However, we observed no differences in the number of newly formed NG2^+^ cells at the level of lateral ventricle (Fig. 5). We also quantified in the same brain regions the number of mature oligodendrocytes using as a marker APC antibodies (directed against the protein adenomatous polyposis coli), but no significant differences were found (data not shown) between the experimental groups.

## Discussion

In the present study, we provide evidence for a proneurogenic effect of FTY720 in vitro, whereby this drug favours the differentiation of postnatal SVZ-derived NSCs into both neurons and oligodendrocytes. In turn, in adult animals, FTY720 increases in the number of new neuroblasts in SGZ following KA-mediated injury. The OPC population is only partially affected by FTY720 treatment, depending on the analyzed regions.

Postnatal and adult NSCs could have different differentiation potentials, although a recent study in vitro reported a similar behaviour of postnatal (p7) and adult (p42)-derived NSCs [28]. Despite this consideration, we decided to use adult animals for the in vivo protocol as the main aim of our study was to examine the response of neurogenic niches to FTY720 after injury.

KA-induced status epilepticus (SE) is an extensively used seizure model of temporal lobe epilepsy in rodents and results primarily in a reproducible pattern of excitotoxic hippocampal cell death [35, 36], as well as in a robust neuroinflammatory response and gliosis [37, 38]. KA-induced seizures in rodents also increase neurogenesis in DG, but in an aberrant and possibly pathogenic way [39]. In fact, KA-induced neurogenesis is accompanied by immature neurons and granule cell dispersion within the adult GCL, widening of the hippocampal GCL, abnormal morphology of newborn neurons and mossy fiber sprouting, abnormal connectivity of newborn neurons into the hippocampal circuits [2, 31, 40–43]. Increase of neurogenesis after SE is considered predominantly detrimental, contributing to epileptogenesis and chronic epilepsy [31], as well as to NSCs pool depletion in SGZ [44]. On the other hand, aging affects hippocampal neurogenic response to seizures with a shift toward glial differentiation, and severe cognitive deficits and chronic epilepsy transformation [45, 46]. Interestingly, SE-induced epileptogenesis is not disrupted in cyclin D2 knockout mice which has a markedly reduced adult neurogenesis [47]. Therefore, the functional relevance of increased neurogenesis after seizure remains doubtful [31, 48] though it is assumed that the neurogenic response after acute seizure activity occurs as an endogenous repair mechanism.

In this study, we observed an increase of proliferating cells after KA-induced injury which was not modulated by FTY720. Interestingly, FTY720 favours a neuronal fate choice of newly born cells, as a significant number of BrdU^+^ cells are also DCX^+^. Thus, FTY720 redirects endogenous neurogenesis to a neuronal fate which may contribute to the previously observed improved neuronal outcome in this injury paradigm [32]. These findings are in line with those showing enhanced hippocampal neurogenesis and memory after chronic administration with FTY720 in adult mice [19, 20] and in mice subjected to chronic unpredictable stress, an experimental paradigm of depression [22]. However, further analysis will be require to verify morphology, integration in GCL, and functionality of new neuroblasts in FTY720-treated animals in our experimental conditions and at longer time points after injury. The use of later markers for fully mature neurons (e.g. NeuN) in combination with BrdU staining would allow to determine whether the neurogenic potential of FTY720 is long-lasting and whether DCX-positive cells can exhibit long-term survival in the SGZ–GCL and constitute a proportion of mature granule cells. FTY720 also reduces microgliosis and brain inflammation after KA-induced SE [32, 49]. Pro-inflammatory microglia have a negative impact on neurogenesis [50, 51], thus FTY720, modulating the inflammatory response, may exert its proneurogenic activity modifying the microenvironmental milieu after acute injury.

Mobilization of OPC population after demyelinating injury represents an attractive therapeutic strategy to support remyelination and consequently brain function. Zhang and colleagues recently demonstrated that FTY720 promotes proliferation and differentiation of OPCs in EAE mice model, in the SVZ, striatum, corpus callosum, and white matter spinal cord, by activating pathways of oligodendrogenesis [14]. OPCs and oligodendrocytes are susceptible to excitotoxic damage, and myelin degeneration has been observed at various time points and in different brain regions after KA-induced neuronal death [52]. Therefore, KA-induced acute injury represents a good model to test the oligodendrogenic effect of FTY720, and as previous studies have demonstrated how pilocarpine-induced SE increases SVZ gliogenesis and attracts newly generated glia to regions of hippocampal damage [53]. Nevertheless, in our experimental conditions, after treatment with FTY720, we found a small, though significant, change in the number of newly generated OPC/NG2 cells in the analyzed area. Thus, in the corpus callosum at the level of dorsal ipsilateral hippocampus, near the most profusely damaged area, we observed a significant increase of newly generated NG2^+^ cells in KA + FTY720-treated group compared to KA-treated animals, and the effect was maintained when we pooled together data from all analyzed regions. However, the effect was lost at more distal white matter regions, such as the corpus callosum at the level of lateral ventricles. Additional studies are needed to better understand the role of FTY720 on oligodendrogenesis in pathological conditions, mostly at later disease stages.

Alternatively, to enhance repair by endogenous NSCs, neuronal or oligodendrocyte lost after disease can be replaced by engraft of exogenous stem cells [54]. However, this therapeutic strategy may be limited due to cell death of transplanted cells, rejection of donor cells and tumorigenesis [55]. In our study, we demonstrated the ability of the pro-drug FTY720, in its non-phosphorylated form, to promote differentiation of postnatal NSCs into both neurons and oligodendrocytes; therefore, FTY720 may be a promising drug for the manipulation of NSC pool used in stem cells graft therapies, promoting their survival, proliferation and oriented differentiation, as well as integration [17, 18]. The active form of FTY720 is considered the phosphorylated one; however, a growing literature demonstrates the effectiveness of the non-phosphorylated form of FTY720 not only in vivo, where FTY720 can be phosphorylated by endogenous sphingosine kinase 2, but also in vitro [17, 18, 20, 32, 56–58]*.* In our NSC cultures, we cannot exclude that the effect observed with FTY720 is due to phosphorylation FTY720-P by sphingosine kinases eventually present in the cultures, as reported elsewhere [17, 58]. In effect, experiments performed with FTY720-P are in support of this hypothesis (Additional file 1: Figure S1), that is the effect we showed in Fig. 1 is due to S1P receptors modulation.

## Conclusions

In summary, we provided evidence of a neurogenic and oligodendrogenic role of FTY720 in vitro, on SVZ-derived NSCs. The efficacy of FTY720 in inducing differentiation of NSCs was partially maintained also in vivo, as we showed an increase in DCX expressing new neurons in SGZ both in basal conditions and in rats after KA-induced damage. Conversely, FTY720 had only partial effect on oligodendrogenesis in this experimental model. These results support a modulatory effect of FTY720 on the SVZ and SGZ neurogenic niches which in the later may foster endogenous repair mechanisms of the adult hippocampal region after pathological insults.

## Additional files

Additional file1: Figure S1. Neurosphere culture differentiation into neurons and oligodendrocytes. Dose-response for FTY720-P treatment and immunofluorescence on fixed cells was performed using anti-β-III tubulin or CNPase as markers of mature neurons or oligodendrocytes, respectively, (representative photomicrographs of the staining are shown in the upper panel of the figure). Total nuclei were stained with DAPI. Fluorescence intensity both from neurons (β-III tubulin) or oligodendrocytes (CNPase), and total nuclei (DAPI) were measured using a fluorescence plate reader with appropriate excitation and emission filters. Differentiation was evaluated as a ratio of β-III tubulin or CNPase fluorescence intensity over total nuclei fluorescence intensity (graphs in the lower panel of the figure). The data are expressed as mean ± SEM of four independent experiments. Statistical analysis: two tailed Student t test; *p < 0.05 and **p < 0.01 vs control. (TIFF 4858 kb)

Additional file 2: Figure S2. Immunostaining with Ki67 showed no relevant difference with BrdU staining*.* (**a**) Representative images of immunoperoxidase staining of Ki67 in ipsilateral and contralateral DG in all experimental groups. Scale bar: 200 μm. DG is surrounded in white dotted line. GCL and SGZ are indicated with arrows. Arrowheads indicate the magnified view in the box. (**b**) Quantitative analysis of the immunoperoxidase staining for Ki67 in ipsilateral and contralateral SGZ of the dorsal hippocampus in control, FTY720, KA and KA + FTY720-treated animals. Positive cells were considered to be within the SGZ if they were within two cell body diameters of the border between the GCL and the hilus. Data were plotted as the mean of Ki67^+^ cells per slice ± SEM. Statistical analysis: Mann Whitney test; *p < 0.05 vs control. Two-way ANOVA showed no significant difference between ipsilateral and contralateral Ki67^+^ cells in SGZ. *N* = 4 for each experimental group. (TIFF 3666 kb)

Additional file 3: Figure S3. Intraperitoneal treatment with FTY720 increases the number of new DCX-positive cells in the SGZ. Animals were sacrificed 8 days after surgery, and 40-μm-thick coronal slices of the dorsal hippocampus were obtained. Double immufluorescence staining for BrdU and DCX was then performed in ipsilateral DG in vehicle (control), FTY720 (ip), KA (icv) and KA (icv) + FTY720 (ip) injected animals. BrdU and DCX-positive cells were counted using confocal acquired images over the total SGZ of the ipsi DG in two sections per animal, corresponding to two levels of the dorsal hippocampus. DCX and BrdU colocalization was determined examining three-dimensional orthogonal reconstructions of confocal layers (ImageJ software). Data are presented as (a, b) mean of positive cells per slice ± SEM and (c) percentage of DXC+ cells over BrdU+ total cells. Statistical analysis: one-way ANOVA followed by Bonferroni post hoc test; *p < 0.05, **p < 0.01. The number of animals per group: control = 3, FTY720 = 3, KA = 2, KA + FTY720 = 2. (TIFF 410 kb)

## Abbreviations

ANOVA: Analysis of variance
BBB: Blood-brain barrier
BDNF: Brain-derived nerve factor
bFGF: Basic fibroblast growth factor
BSA: Bovine serum albumin
BrdU: 5-Bromo-2-deoxyuridine
CC: Corpus callosum
CNPase: 2′,3′-Cyclic nucleotide 3′-phosphodiesterase
CNS: Central nervous system
CNTF: Ciliary neurotrophic factor
DAB: 3,3′-Diaminobenzidine
DG: Dentate gyrus
DIV: Days in vitro
DMEM: Dulbecco’s Modified Eagle Medium
DMSO: Dimethyl sulfoxide
DCX: Doublecortin
EAE: Experimental autoimmune encephalomyelitis
EGF: Epidermal growth factor
GCL: Granular cell layer
*icv*: Intracerebroventricular
*ip*: Intraperitoneally
KA: Kainic acid
LPC: Lysolecithin
MS: Multiple sclerosis
NGF: Nerve growth factor
NG2: Chondroitin sulphate proteoglycan neuron/glia antigen 2
NPCs: Neural progenitor cells
NSCs: Neural stem cells
NT3: Neurothrophin-3
OPCs: Oligodendrocyte progenitor cells
PBS: Phosphate buffered saline
PEDF: Pigment epithelium-derived factor
PI: Propidium iodide
SE: Status epilepticus
S1P: Sphingosine-1-phosphate
S1PRs: S1P receptors
SGZ: Subgranular zone
SVZ: Subventricular zone
