# Supplementary figures and images for: Effects of FTY720 on brain neurogenic niches in vitro and after kainic acid-induced injury
Source: J Neuroinflammation. 2017 Jul 24;14:147. doi: 10.1186/s12974-017-0922-6 (PMC5525223; doi:10.1186/s12974-017-0922-6)

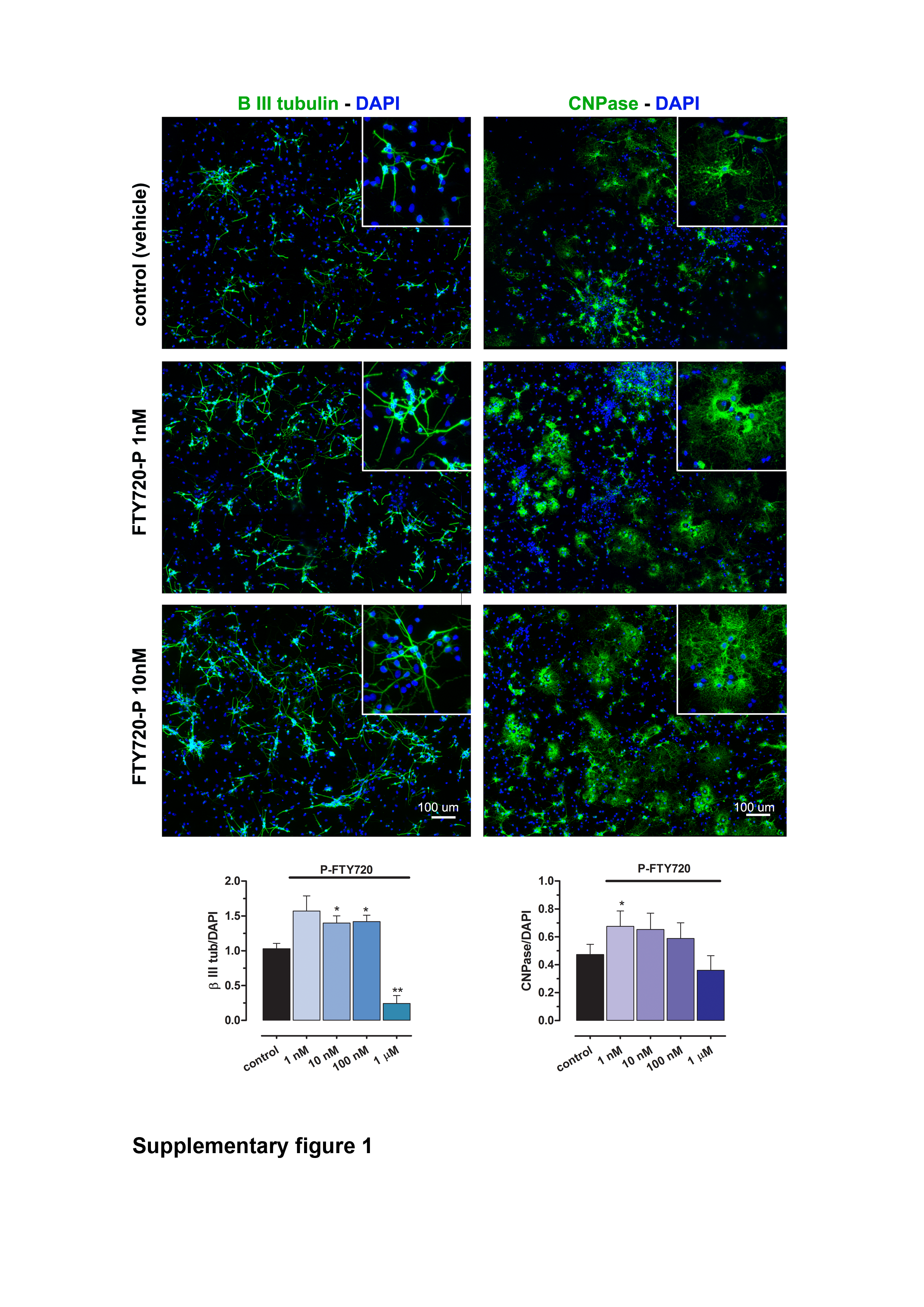

Supplement: Supplementary file 1 — Neurosphere culture differentiation into neurons and oligodendrocytes. Dose-response for FTY720-P treatment and immunofluorescence on fixed cells was performed using anti-β-III tubulin or CNPase as markers of mature neurons or oligodendrocytes, respectively, (representative photomicrographs of the staining are shown in the upper panel of the figure). Total nuclei were stained with DAPI. Fluorescence intensity both from neurons (β-III tubulin) or oligodendrocytes (CNPase), and total nuclei (DAPI) were measured using a fluorescence plate reader with appropriate excitation and emission filters. Differentiation was evaluated as a ratio of β-III tubulin or CNPase fluorescence intensity over total nuclei fluorescence intensity (graphs in the lower panel of the figure). The data are expressed as mean ± SEM of four independent experiments. Statistical analysis: two tailed Student t test; *p < 0.05 and **p < 0.01 vs control. (TIFF 4858 kb) [file 12974_2017_922_MOESM1_ESM.tif]

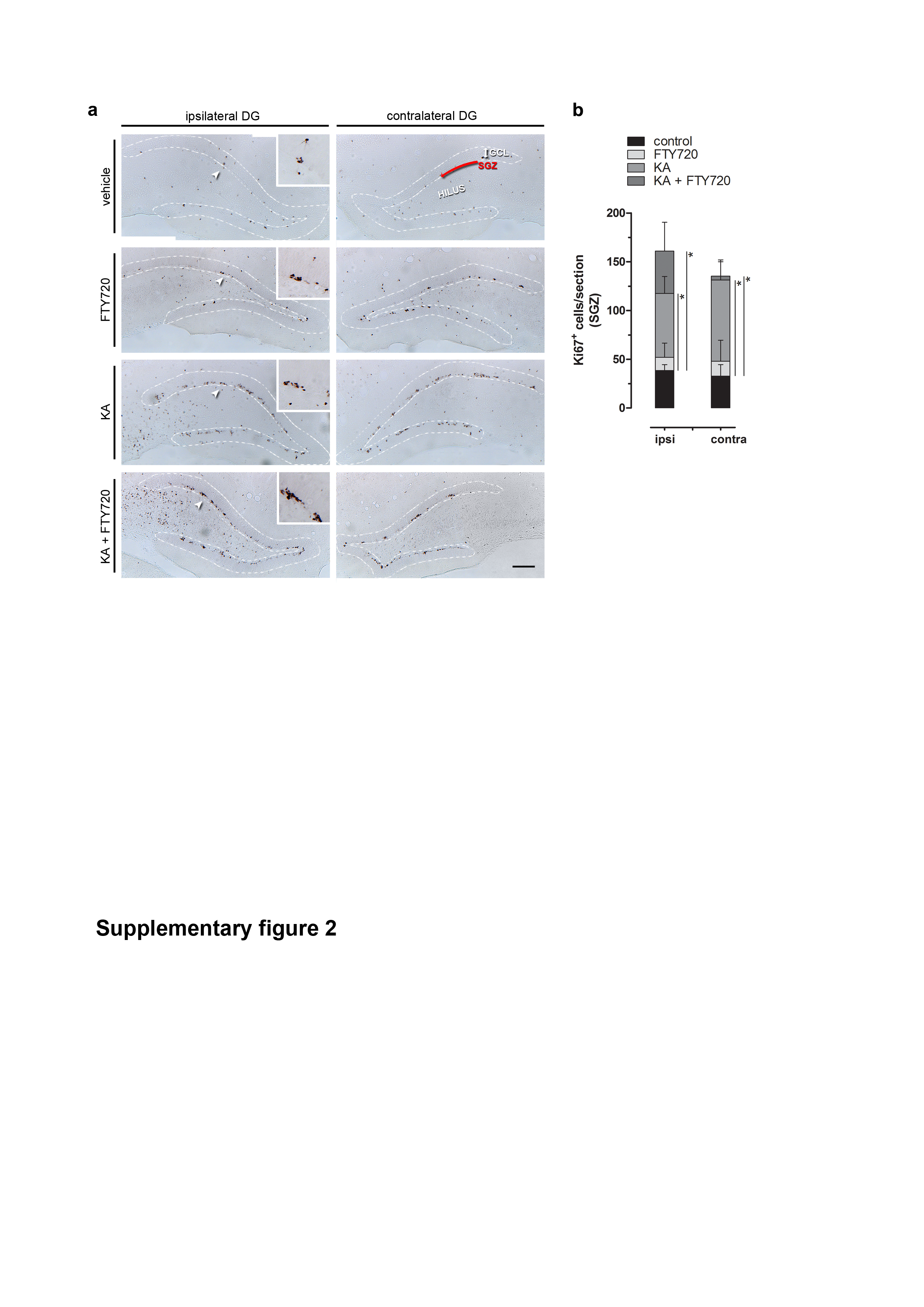

Supplement: Supplementary file 2 — Immunostaining with Ki67 showed no relevant difference with BrdU staining. (a) Representative images of immunoperoxidase staining of Ki67 in ipsilateral and contralateral DG in all experimental groups. Scale bar: 200 μm. DG is surrounded in white dotted line. GCL and SGZ are indicated with arrows. Arrowheads indicate the magnified view in the box. (b) Quantitative analysis of the immunoperoxidase staining for Ki67 in ipsilateral and contralateral SGZ of the dorsal hippocampus in control, FTY720, KA and KA + FTY720-treated animals. Positive cells were considered to be within the SGZ if they were within two cell body diameters of the border between the GCL and the hilus. Data were plotted as the mean of Ki67+ cells per slice ± SEM. Statistical analysis: Mann Whitney test; *p < 0.05 vs control. Two-way ANOVA showed no significant difference between ipsilateral and contralateral Ki67+ cells in SGZ. N = 4 for each experimental group. (TIFF 3666 kb) [file 12974_2017_922_MOESM2_ESM.tif]

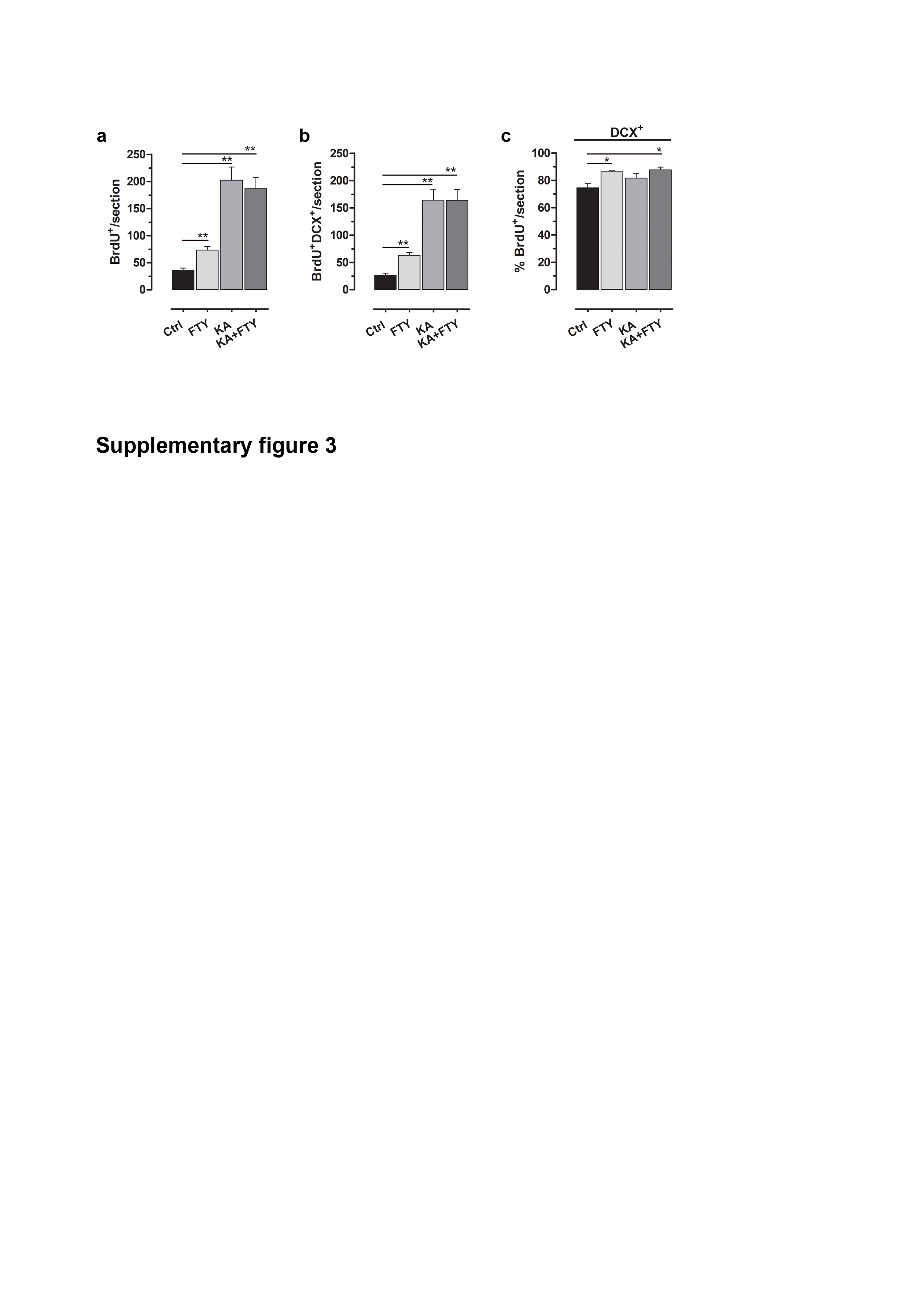

Supplement: Supplementary file 3 — Intraperitoneal treatment with FTY720 increases the number of new DCX-positive cells in the SGZ. Animals were sacrificed 8 days after surgery, and 40-μm-thick coronal slices of the dorsal hippocampus were obtained. Double immufluorescence staining for BrdU and DCX was then performed in ipsilateral DG in vehicle (control), FTY720 (ip), KA (icv) and KA (icv) + FTY720 (ip) injected animals. BrdU and DCX-positive cells were counted using confocal acquired images over the total SGZ of the ipsi DG in two sections per animal, corresponding to two levels of the dorsal hippocampus. DCX and BrdU colocalization was determined examining three-dimensional orthogonal reconstructions of confocal layers (ImageJ software). Data are presented as (a, b) mean of positive cells per slice ± SEM and (c) percentage of DXC+ cells over BrdU+ total cells. Statistical analysis: one-way ANOVA followed by Bonferroni post hoc test; *p < 0.05, **p < 0.01. The number of animals per group: control = 3, FTY720 = 3, KA = 2, KA + FTY720 = 2. (TIFF 410 kb) [file 12974_2017_922_MOESM3_ESM.tif]
